# Copy number variation in human genomes from three major ethno-linguistic groups in Africa

**DOI:** 10.1186/s12864-020-6669-y

**Published:** 2020-04-10

**Authors:** Oscar A. Nyangiri, Harry Noyes, Julius Mulindwa, Hamidou Ilboudo, Justin Windingoudi Kabore, Bernardin Ahouty, Mathurin Koffi, Olivier Fataki Asina, Dieudonne Mumba, Elvis Ofon, Gustave Simo, Magambo Phillip Kimuda, John Enyaru, Vincent Pius Alibu, Kelita Kamoto, John Chisi, Martin Simuunza, Mamadou Camara, Issa Sidibe, Annette MacLeod, Bruno Bucheton, Neil Hall, Christiane Hertz-Fowler, Enock Matovu, for the TrypanoGEN Research Group, as members of The H3Africa Consortium

**Affiliations:** 1 College of Veterinary Medicine, Animal Resources and Biosecurity, Makerere University, P. O. Box 7062, Kampala, Uganda; 2 Epidemiology and Demography Department, Kenya Medical Research Institute (KEMRI)/Wellcome Trust Research Programme, P.O. Box 230, Kilifi, Kenya; 3 Centre for Genomic Research, University of Liverpool, Liverpool, L69 7ZB UK; 4 Institut de Recherche en Sciences de la Santé (IRSS) - Unité de Recherche Clinique de Nanoro (URCN), Nanoro, Burkina Faso; 5 Centre International de Recherche-Développement sur l’Elevage en zones Subhumides (CIRDES), Unité des Maladies à Vecteurs et Biodiversité (UMaVeB), 01 BP 454, Bobo-Dioulasso, 01 Burkina Faso; 6 Felix Houphouet Boigny University (UFHB), Cocody, Abidjan, Côte d’Ivoire; 7 Université Jean Lorougnon Guédé (UJLoG) de Daloa, Daloa, Côte d’Ivoire; 8 Institut National de Recherche Biomedicale, Avenue de la Democratie, Kinshasa Gombe, P. O. Box 1197, Kinshasa, Democratic Republic of Congo; 9 Faculty of Science, University of Dschang, P. O. Box 67, Dschang, Cameroon; 10 College of Natural Sciences, Makerere University, P. O. Box 7062, Kampala, Uganda; 11 College of Medicine, Department of Basic Medical Sciences, University of Malawi, Private Bag 360, Chichiri, Blantyre, 3 Malawi; 12 Department of Disease Control, School of Veterinary Medicine, University of Zambia, P. O. Box 32379, Lusaka, Zambia; 13 Programme National de Lutte contre la Trypanosomose Humaine Africaine, BP 851, Conakry, Guinea; 14 Wellcome Centre for Molecular Parasitology, Institute of Biodiversity, Animal Health and Comparative Medicine, Garscube Estate, Glasgow, G61 1QH UK; 15 Institut de Recherche pour le Développement (IRD), IRD-CIRAD 177, TA A-17/G, Campus International de Baillarguet, F-34398 Montpellier, France; 16 Present address: Earlham Institute Norwich Research Park Innovation Centre, Colney Ln, Norwich, NR4 7UZ UK

**Keywords:** CNV, Structural variation, Niger Congo A, Niger Congo B, Nilo-Saharan, Signatures of selection, Adaptation, Tag haplotypes

## Abstract

**Background:**

Copy number variation is an important class of genomic variation that has been reported in 75% of the human genome. However, it is underreported in African populations. Copy number variants (CNVs) could have important impacts on disease susceptibility and environmental adaptation. To describe CNVs and their possible impacts in Africans, we sequenced genomes of 232 individuals from three major African ethno-linguistic groups: (1) Niger Congo A from Guinea and Côte d’Ivoire, (2) Niger Congo B from Uganda and the Democratic Republic of Congo and (3) Nilo-Saharans from Uganda. We used GenomeSTRiP and cn.MOPS to identify copy number variant regions (CNVRs).

**Results:**

We detected 7608 CNVRs, of which 2172 were only deletions, 2384 were only insertions and 3052 had both. We detected 224 previously un-described CNVRs. The majority of novel CNVRs were present at low frequency and were not shared between populations. We tested for evidence of selection associated with CNVs and also for population structure. Signatures of selection identified previously, using SNPs from the same populations, were overrepresented in CNVRs. When CNVs were tagged with SNP haplotypes to identify SNPs that could predict the presence of CNVs, we identified haplotypes tagging 3096 CNVRs, 372 CNVRs had SNPs with evidence of selection (iHS > 3) and 222 CNVRs had both. This was more than expected (*p* < 0.0001) and included loci where CNVs have previously been associated with HIV, Rhesus D and preeclampsia. When integrated with 1000 Genomes CNV data, we replicated their observation of population stratification by continent but no clustering by populations within Africa, despite inclusion of Nilo-Saharans and Niger-Congo populations within our dataset.

**Conclusions:**

Novel CNVRs in the current study increase representation of African diversity in the database of genomic variants. Over-representation of CNVRs in SNP signatures of selection and an excess of SNPs that both tag CNVs and are subject to selection show that CNVs may be the actual targets of selection at some loci. However, unlike SNPs, CNVs alone do not resolve African ethno-linguistic groups. Tag haplotypes for CNVs identified may be useful in predicting African CNVs in future studies where only SNP data is available.

## Background

Copy number variants are defined as duplications or deletions of genomic segments greater than 1 kb in length [1]. While most genomic studies focus on single nucleotide variants (SNV), reports of larger genomic variants such as copy number variants (CNVs) are more limited [2]. However, given their size, CNVs cover more bases than SNV [2] and may have greater influence on gene expression and structure [3, 4]. These variations can also be associated with disease or adaptations to changing environments [5–7]. In addition, CNVs can be the functional variant underlying quantitative trait loci (QTL) found by genome wide association studies (GWAS).

African populations have the highest genomic diversity globally [8]. The four major ethno-linguistic groups in Africa are the Afro-Asiatic, Nilo-Saharan, Khoisan and Niger Congo, the latter of which consists of two major subdivisions; Niger-Congo-A and Niger-Congo-B [9]. These populations occupy diverse environments, have different cultures and ancestry and show stratification at genomic level [9]. Such genomic differences between groups may be associated with differences in susceptibility to infectious diseases such as malaria, tuberculosis and HIV [10] or environmental adaptations such as increases in copies of amylase genes associated with increased carbohydrate consumption [5, 11]. Studies of genomic variation such as CNVs in Africans may therefore help explain adaptation, population stratification and disease susceptibility.

African populations are under-represented in genomic studies [12], but are likely to harbour a large number of unique CNVs given their higher genomic diversity than European, American and Asian populations [8]. Here, we analyse whole genome sequence (WGS) data for CNVs in populations from Nilo-Saharan, Niger Congo A and Niger Congo B ethno-linguistic groups. Niger Congo A and Niger Congo B are the two largest linguistic groups in Africa. Niger Congo B is comprised of the Bantu languages and is a subgroup of Niger Congo A and therefore these two groups are a single lineage. We included the Nilo-Saharan Lugbara as an out group to make it possible to contrast diversity within the Niger-Congo populations with diversity between major linguistic groups.

The populations surveyed and their respective countries were: Ugandan Nilo-Saharans of Lugbara ethnicity (UNL, *n* = 50); Niger-Congo-B speaking populations from Uganda (UBB, *n* = 33) and the Democratic Republic of Congo (DRC, n = 50); and Niger-Congo A speaking populations from Côte d’Ivoire (CIV, n = 50) and Guinea (GAS, *n* = 49). We aimed to discover novel CNV region (CNVR) variants, investigate population differences associated with CNVs and identify SNP haplotypes which tag CNVs and may predict such CNVs in future genome wide association studies (GWAS). The CNVs identified may also be important in understanding African CNV diversity and allowing inference of CNVs from population specific SNP-chip data.

## Results

### Participant characteristics

The countries of origin and ethnicities of participants are shown in Table 1 and a full list of the 232 samples is shown in Additional file 1. We used about 50 samples per population except for 33 from the Ugandan UBB population (Table 1). 50 samples provide a 95% chance of discovering CNVRs that have a frequency greater than 7%, while 232 samples give a 95% chance of detecting CNV with greater than 2% frequency.

**Table 1:** Ethnicity and origin of individuals analysed for CNV

Pop	Country	District	Ethno-linguistic group (ethnologue code, n)
UNL	Uganda	Maracha	Lugbara (IGG, 50)
UBB	Uganda	Iganga	Basoga (XOG, 33)
DRC	Democratic Republic of Congo	Bandundu	Kingongo (NOQ, 30) Kimbala (MDP, 20)
GAS	Guinea	Forecariah Boffa, Dubreka	Soussou (SUS, 49)
CIV	Côte d’Ivoire	Bonon Sinfra	Baoule (BCI, 11) Gouro (GOA 21) Moore (MOS, 12) Senoufo (SEF, 4) Malinke (LOI, 1) Koyaka (KGA, 1)

Ethnologue codes are derived from the ethnic languages of the world resource [13]

### Identification of CNVs

To examine the distribution and extent of CNVs in human African populations, we selected 232 individuals from four countries (Table 1), representing Ugandan Nilo-Saharan population of Lugbara ethnicity (UNL); Niger-Congo B-speaking populations from Uganda (UBB) and Democratic Republic of Congo (DRC); Niger Congo A speakers from Côte d’Ivoire (CIV) and Guinea (GAS). Mean depth of sequence coverage was 10X and we used autosomal data only. We used two programs adapted for population scale data for CNV discovery: cn.MOPS and GenomeSTRiP, which have been benchmarked previously (see Materials and Methods). cn.MOPS calls CNVs based on read depth alone, whereas GenomeSTRiP combines read pairs, split reads, and read depth to generate CNV calls [14].

### Comparison of cn.MOPS and GenomeSTRiP

**Fig. 1:**
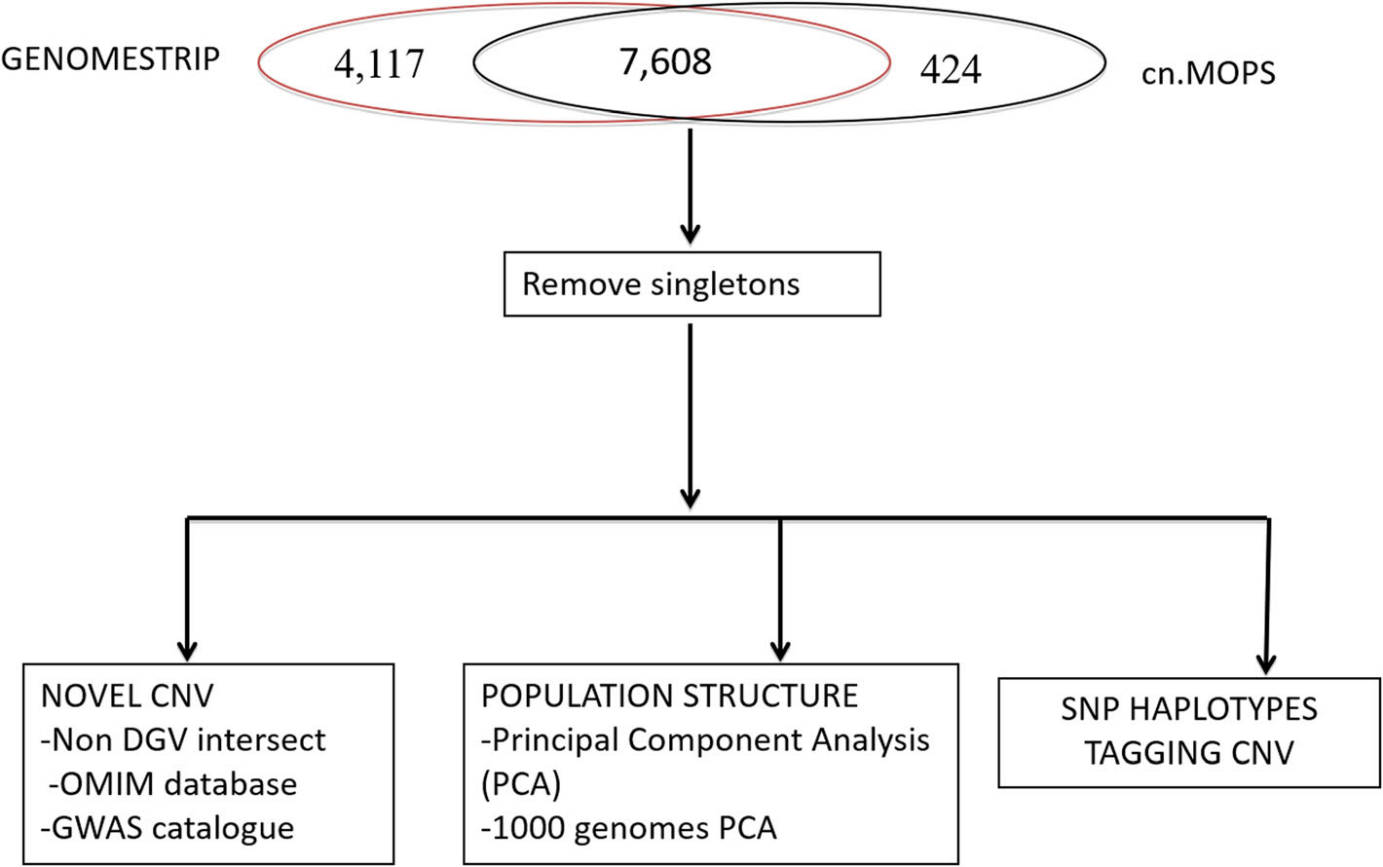
Selection of high confidence CNV and analysis strategy. GenomeSTRiP CNVR overlapping cn.MOPS CNVR were selected and singletons assessed for removal. The resulting consensus dataset was annotated to identify novel CNVs, show population structure deduced from CNV calls and tag SNP analysis

Figure 1 summarizes the analysis workflow and Table 2 shows descriptive statistics for the CNVs predicted by the two methods. Additional file 2 and Figs S1 A & B give further details on comparison of CNV called by both methods. GenomeSTRiP detected 16,149 CNVRs compared to 9213 detected by cn.MOPS. The CNVR were filtered by removing 37 samples that appeared to be outliers on a multiple dimensional scaling plot (MDS) (Additional file 2: Fig S2). These outlier samples all had exceptionally high numbers of CNVRs, mean of outliers = 2718 compared with mean of retained = 548, *p* = 6.4e-09 and also had higher inbreeding co-efficient (F) [15], F = 0.13 for outliers compared with F = 0.04 for non-outliers, *p* = 7.8e-05.

**Table 2:** CNV statistics using GenomeSTRiP and cn.MOPS algorithms

Parameter	GenomeSTRiP	cn.MOPS	GenomeSTRiP that overlap cn.MOPS
Raw CNV regions (CNVR)	16,149	9213	
CNVR after QC	11,275	2115	7608
Total CNV scored	127,699	37,679	106,922
Deletion CNV	65,588	26,008	61,025
Gain CNV	62,111	11,671	45,897
Mean CNV count per CNVR	11.3	17.8	14.0
Mean CNVR per individual	654	193	548
Count of overlapping CNVRs ^a^	7608	1691	7608
Mean Length of CNVR (kb)	9.5	541.7	10.7
SD length of CNVR (kb)	13.2	1287.6	14.1
Median Length of CNVR (kb)	5.3	32.4	6
Total Length of CNVR (Mb)	108.1	1145.8	81.2
Observed Length CNV present in both methods (Mb) (Simulated ± SD)^b^	81.2 (43.4 ± 1.0)		

Descriptive statistics of CNVR found using GenomeSTRiP and cn.MOPS. Note that: GenomeSTRiP has about 5.3 times the number of CNVs compared with cn.MOPS (11,275 cf. 2115); GenomeSTRiP CNVRs were shorter (median length 5.3 kb) than cn.MOPS (median length 32.4 kb); Total length of cn.MOPS CNVRs was about 10.6 times greater (1146 Mb cf. 108 Mb) than GenomeSTRiP CNVRs. CNVR = CNV region; a genomic location with chromosome, start and end base pair positions that has overlapping CNVs; CNVRs after QC = The CNVRs left after some CNVRs were dropped because they were only found in samples that were outliers in principal component analysis (PCA) plots of raw data. CNV count per CNVR = Number of samples with a CNV at each CNV region = Total CNVs count/ Total CNVRs; Mean CNVRs per sample = Count of CNV divided by number of samples; Mean, Standard deviation, Median, Total length, Observed length: Calculated per CNV not CNVR

^a^Count of any overlap (minimum 1 bp) between GenomeSTRiP and cn.MOPS CNVR

^b^The expected length of CNVs that would be found by both methods was obtained by 100 simulations using all the observed lengths of CNVs allocated to random places in the genome

After removing the outliers, predicted CNVR retained for further analysis were 11,725 from GenomeSTRiP and 2115 from cn.MOPS. We defined as high confidence CNVRs those called by both GenomeSTRiP and cn.MOPS. This identified 7608 GenomeSTRiP CNVR that overlapped or were within cn.MOPS loci (Additional file 3). No CNVRs were predicted in a single sample only.

### Characteristics of CNVRs identified by GenomeSTRiP and cn.MOPS

The CNVRs discovered by GenomeSTRiP (median length 5.2 kb) were much shorter than those discovered by cn.MOPS (median length 32 kb) (Table 2) and were more similar in length to those in the database of genomic variants (DGV; release date 2016-05-15) (median length 3.3 kb for CNVR > 1 kb) [16, 17].

GenomeSTRiP called more CNVRs (7608) than cn.MOPS (1691) and there were multiple GenomeSTRiP CNVRs within each cn.MOPS CNVR. The total lengths of CNVRs were 108 Mb and 1145 Mb in GenomeSTRiP and cn.MOPS, respectively. We found that 81 Mb (75%) of the GenomeSTRiP CNVRs were within cn.MOPS CNVRs, almost twice as much as the 43 Mb (40%) that was expected from random placement of the GenomeSTRiP CNVRs by simulation. Given that the GenomeSTRiP CNVRs conformed most closely in size to those described in DGV we used the GenomeSTRiP CNVRs for subsequent analysis. Amongst the 7608 CNVRs, there were 2172 CNVRs with only deletions, 2384 with only insertions and 3052 with both insertions and deletions. Counts of each class of CNV for each population are shown in Additional file 4.

**Fig. 2:**
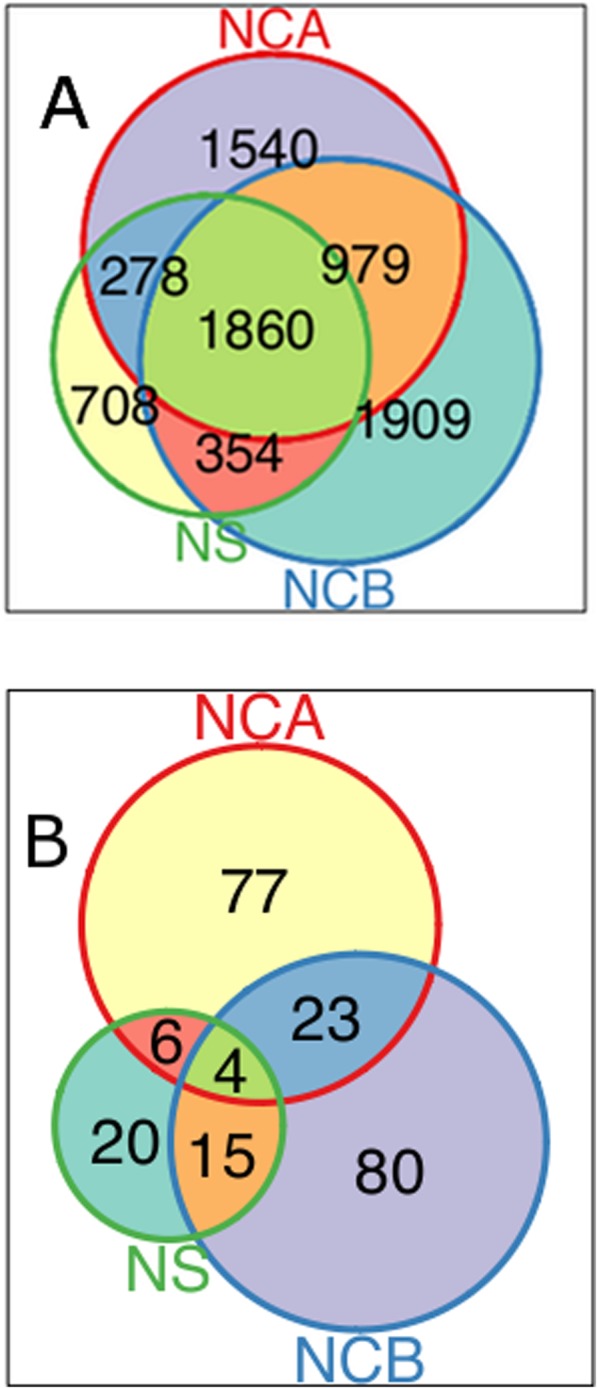
Venn diagram showing counts of CNVR shared between populations. **a** All CNVR from Niger Congo A (NCA), Niger Congo B (NCB) and Nilo-Saharan (NS) ethnic groups. CNVR overlapping 5 kb genomic regions were plotted for each population. A majority of the CNVR are shared between populations, but Nilo-Saharans appear to have the least CNVR, with most of them shared with the Niger Congo A and Niger Congo B. **b** Sharing of novel CNV regions between populations. Most novel CNVR are unique to individual populations studied whereas others are shared. To enable comparison, the genome was divided into 5 kb regions and regions with novel CNVR in each of these regions for each population were compared for overlaps

24% of CNVRs were common to all three major linguistic groups represented in the data, 55% were unique to single linguistic groups and 21% were shared between pairs of major populations (Fig. 2a). Frequencies of shared CNVs were most correlated between Niger-Congo A and Niger-Congo (r^2^ = 0.38), and least correlated between Niger-Congo and Nilo-Saharan (r^2^ = 0.17). Individuals of Nilo-Saharan origin had the lowest proportion of private CNVRs (20%) whilst the Niger-Congo A and Niger-Congo B populations shared more with each other than with the Nilo-Saharans, consistent with their closer linguistic relationship.

### Genomic distribution of CNVR

**Fig. 3:**
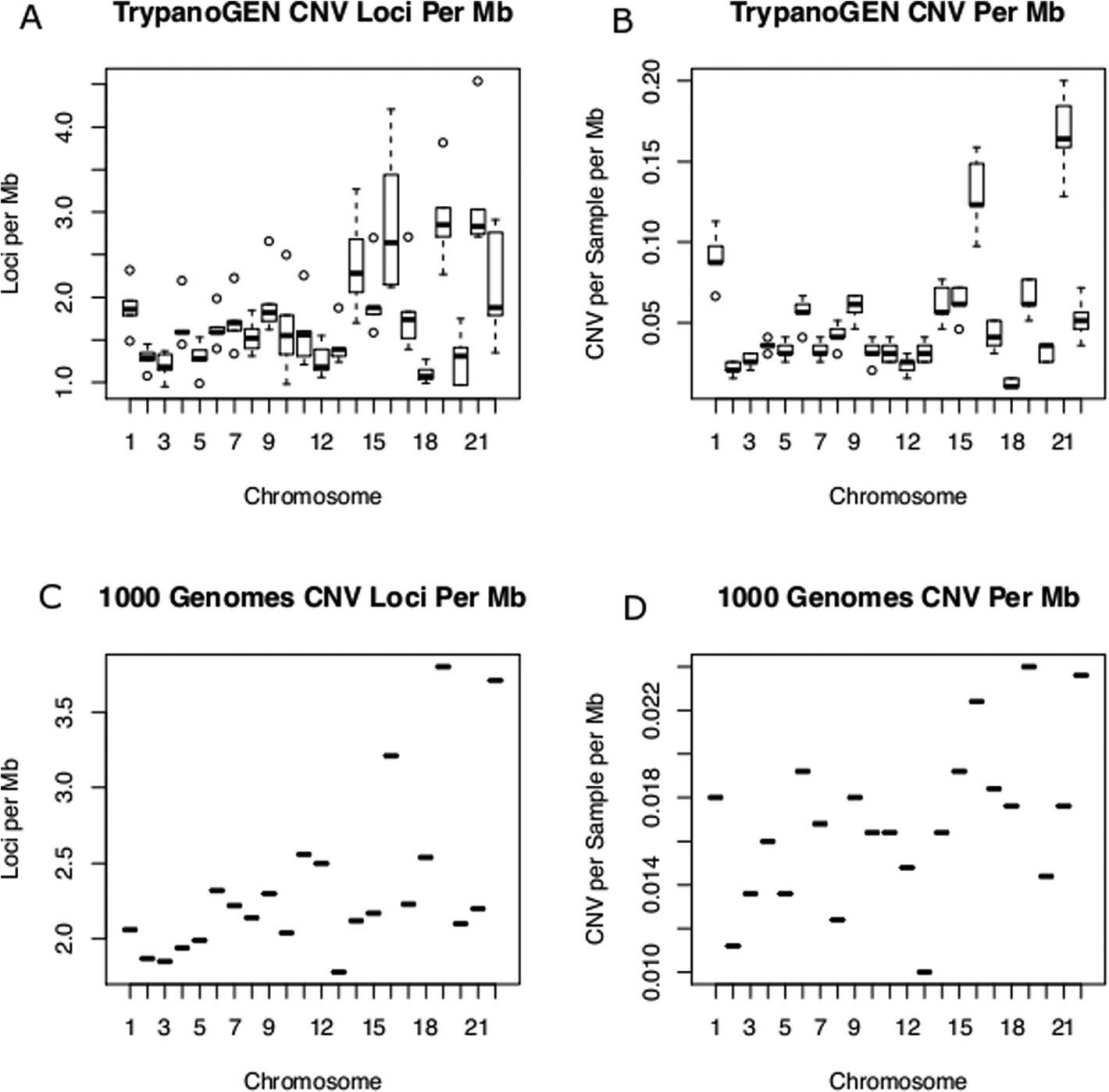
CNV density comparison between TrypanoGEN and the 1000 Genomes project. Counts of Loci per Mb and Counts of CNV per Mb for each chromosome in TrypanoGEN and 1000 Genomes project data. **a** Counts of CNVR per Mb in TrypanoGEN **b** CNV loci counts per Mb in TrypanoGEN **c** Counts of CNVR per Mb in 1000 Genomes **d** CNV loci counts per Mb in TrypanoGEN Both sets show similar patterns of CNV per chromosome, with 1000 Genomes data having tighter interquartile ranges

**Fig. 4:**
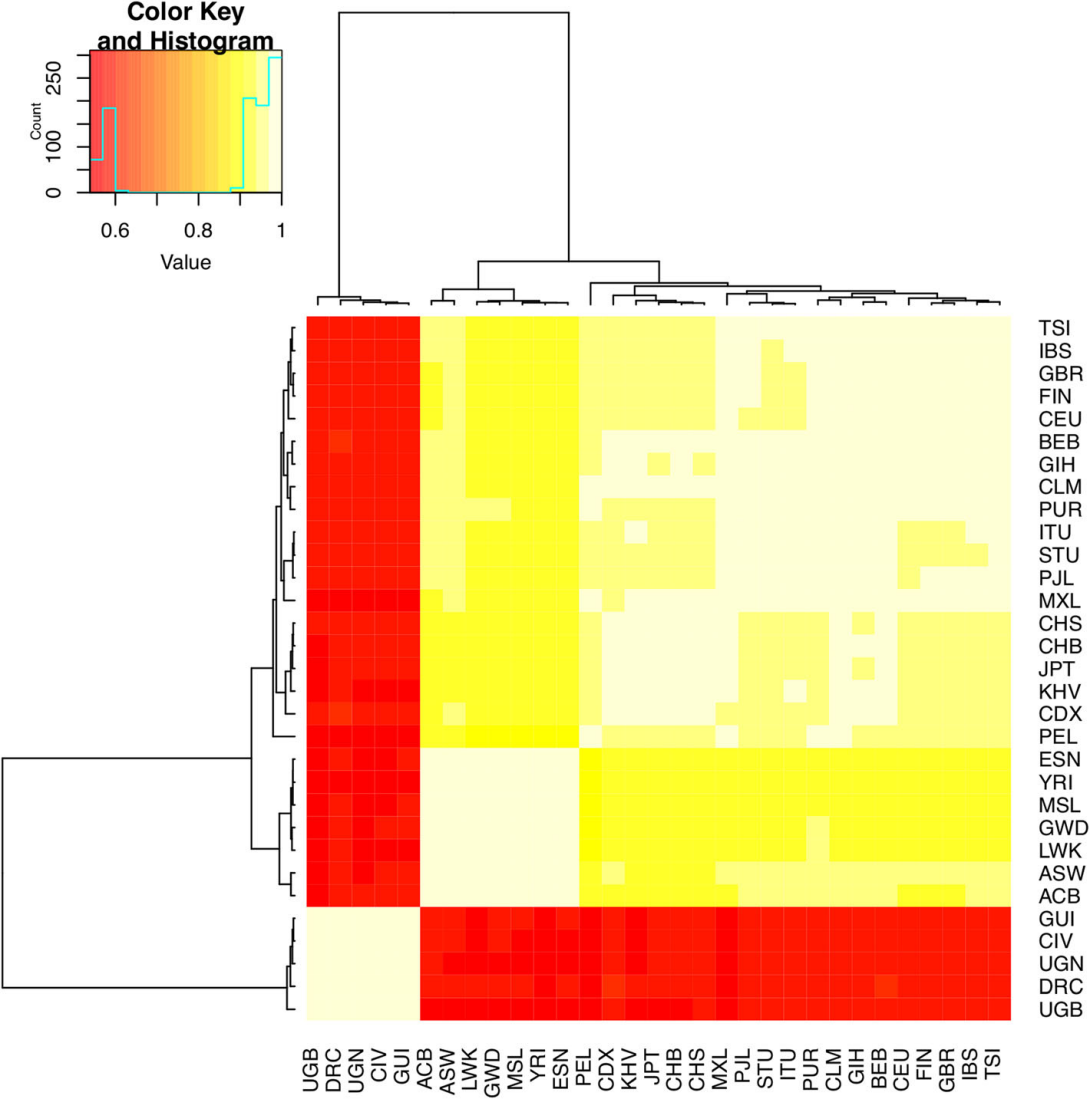
Heat Map showing Pearson Correlation coefficient between the Count of CNV in 10 Mb windows in each population across the genomes of TrypanoGEN and 1000 Genomes samples. The histogram in the legend indicates the number of correlations with each value of Pearson’s r, there are large numbers of correlations between 0.5 and 0.6 and also between 0.9 and 1. Correlation coefficients are high (> 0.9) between populations from the same dataset but lower (0.5–0.6) between populations from different data sets

The density of CNVRs varied by about two-fold (1.43–2.41 CNVRs Mb^− 1^) between the five populations (Additional file 2: Fig S3). The density of CNVRs also varied between chromosomes in both our data and 1000 Genomes data (Fig. 3) with the mean densities per chromosome correlated between both datasets (r^2^ = 0.71) (Fig. 4). The density of CNVs also varied across chromosomes (Additional file 2: Fig S3). The CNVRs per Mb ranged from a minimum of 5 in chromosome 18 to a maximum of 15 in chromosome 21. This trend was similar in counts of CNV calls per Mb with chromosome 18 displaying a minimum of 12 calls and 150 CNVs per Mb predicted on chromosome 21. We tested the 1000 genomes data for CNVR density by chromosome to confirm that variation in CNVR density is common in other datasets. The same phenomenon was observed with chromosomes 19 and 22 having high (~ 24 CNVRs Mb^− 1^) numbers of CNVRs per Mb compared with other chromosomes (~ 14 CNVRs Mb^− 1^) (Fig. 3).

### Functional annotation of CNVR

CNVRs were annotated with the classes of genomic features which they intersected. The most common annotations were coding and open chromatin regions (Additional file 2: Fig S4).

### Novel CNV loci

**Fig. 5:**
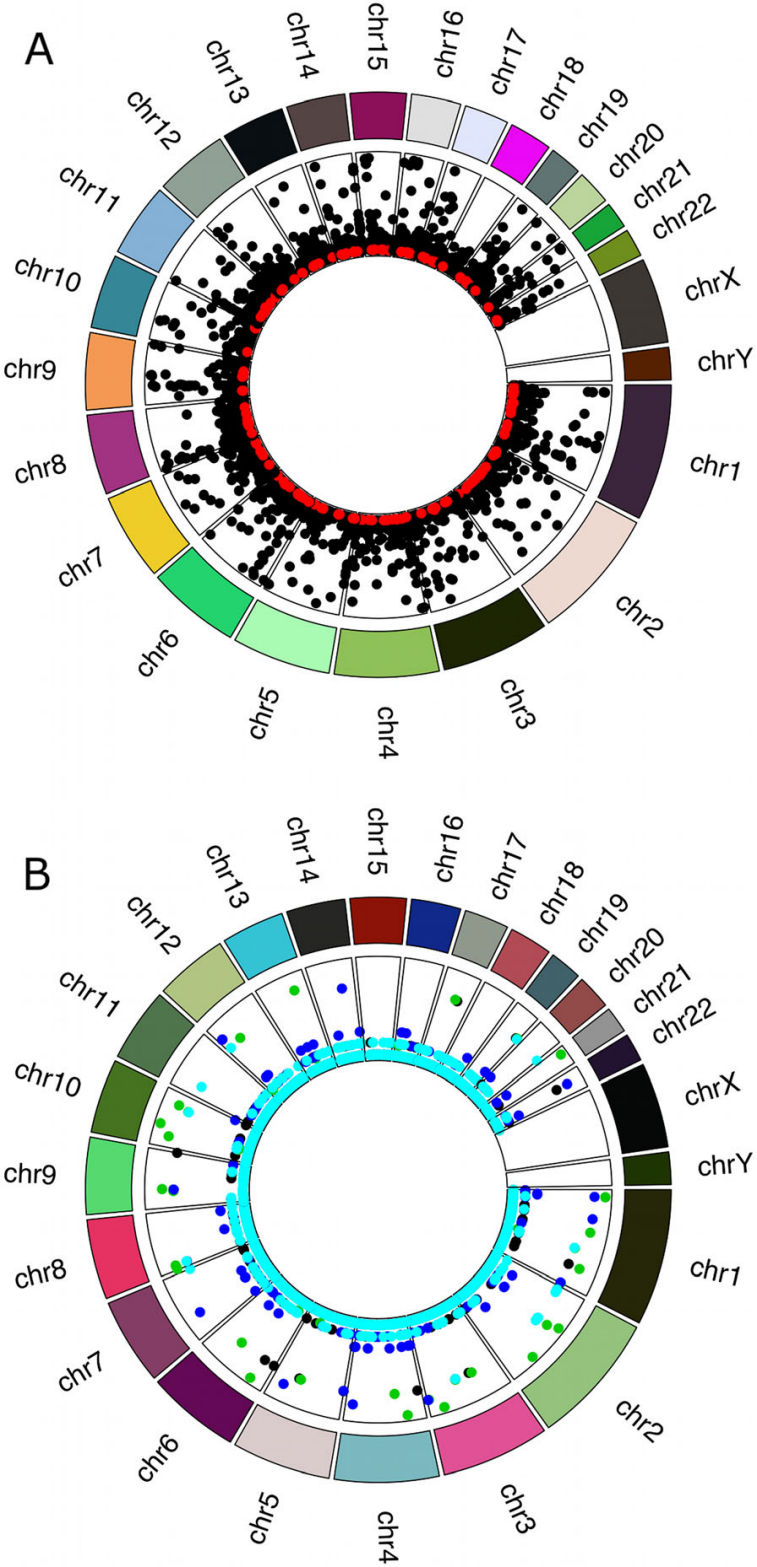
Genomic distribution of CNVR and their frequency in our samples. **a** Known and novel CNVR are distributed throughout the genome, with novel CNVR having lower frequencies compared to known CNVR. The centre of the circle has the least frequency of < 1% whereas the outermost bounds represent higher frequencies of up to 100%. Novel CNVR shown in red are lower frequency compared to known CNVR shown in black. A few known CNVRs show high frequencies. **b** Comparison of frequencies in the various populations. No major differences in CNVR frequencies were found between populations. All populations are represented in the plot with different colours. The centre of the plot has the least frequency of 0% whereas the outermost bounds represent higher CNVR frequencies. Frequencies are similar across populations. The frequencies of CNVRs with CNV frequencies < 20% are set to 0% to enhance visibility. Cyan shows the CNV frequency of those common to GAS and all populations, UBB are in black, DRC are in green, CIV are in dark blue and UGN are in red

We found 7384 of the 7608 final CNVRs analysis set overlapped known CNVRs in the human DGV and 224 (2.9%) had not been previously reported, and were defined as novel CNVRs. Unique CNVR boundaries in the DGV cover 75% of the genome and much of the rest could be repeat regions where reads cannot be mapped with certainty and therefore CNVRs cannot be detected. CNVs in novel CNVRs were 10 times less frequently observed compared with CNV in known CNVR (mean frequency of novel CNVs was 0.74% compared with 7.4% for known CNVs). The novel CNVs were annotated using BEDTools intersect [18] against the list of Ensembl genes and regulatory regions (Additional file 5 and Fig S3B). We sought to clarify the frequency, likely functional roles and sharing of CNVRs between populations. Novel CNVRs were distributed throughout the genome at low frequencies (Fig. 5a). They intersected 293 unique genes or regulatory regions, with no specific function enriched and were not generally shared between the populations (Fig. 2b). When novel CNVRs intersecting protein coding genes were annotated in PANTHER [19] using gene ontology (GO) terms, 27% (30/109) of the novel CNVRs overlapped genes encoding binding function (GO: 0005488) and 20% (22/109) overlapped genes involved in catalytic activity (GO: 0003824). The novel CNVRs also overlap SNPs associated with traits in the genome wide association study catalogue (Additional file 2: Fig S5 and Additional file 6). Using BEDTools intersect; we found that both the known and novel CNVR overlapped Mendelian inheritance disease associated genes (Additional file 7).

### Identification of haplotypes tagging CNVR

SNP haplotypes that tag CNVRs in our populations were identified to assist the interpretation of SNP based GWAS studies. We assumed that if a haplotype is associated with a CNV then the number of alleles (0, 1, 2) of that haplotype will be correlated with the observed number of copies reported in samples in the dataset. Therefore, copy number is plotted against haplotype count for each sample and the value of *r*^*2*^ is calculated for the regression line and also the *p* value that the slope is zero. Haplotype blocks were defined using linkage disequilibrium (r^2^ > 0.8), which has been shown to tag shorter haplotypes in African American genomes compared to West Eurasians [20]. Alleles of 6942 haplotypes were associated with 3096 (41%) CNVRs as shown in Additional file 8. The mean count of CNVs at tagged CNVRs was 27.1 (CNV frequency = 12%) compared with 15.9 (7%) at untagged loci. The proportion of CNVRs that were tagged increased with frequency; less than 36% of CNVRs with CNV frequencies less than 10% were tagged but 64% of CNVRs with frequencies > 10% were tagged (Additional file 2: Fig S6). There was no difference between populations in the proportion tagged. Shorter (< 10 kb) CNVRs were less likely to be tagged (40% tagged) than longer (> 10 kb) CNVRs (49% tagged), reflecting the larger number of haplotypes found in longer CNVRs; there were a mean of 19 haplotypes in CNVRs < 10 kb and 37 haplotypes in CNVRs > 10 kb. Haplotypes that tag the CNVR detected in each of the five populations tested are shown in Additional file 8. The numbers of haplotype tagged CNVRs in each population were; 1286 (38.1%) in the CIV, 1540 (36.6%) in the DRC, 1261 (36.9%) in the GAS, 1169 (40.3%) in the UBB and 3200 (39.0%) in the UNL.

### CNVRs are overrepresented at loci under selection

In order to identify CNVs with potentially functional effects we tested for association between CNVRs and loci that have been identified as under selection, with integrated haplotype score (iHS > 3.0) in the UNL population in a separate study of the same data [21]. There were 12,278 SNPs with evidence of selection (−log10 iHS *p* > 3.0), of these 1805 were within CNVRs, more than twice as many as would be expected by chance (χ = 1822, *p* < 10^− 10^) (Table 3), indicating a positive bias of selection on human CNVRs as shown in a previous study [22].

**Table 3:** Counts of SNPs inside and outside CNVRs with significant (−log10 p > 3) and non-significant *p* values

UNL CNV + 5 kb flanks	-LOG10 *p* > 3	-LOG10 *p* < 3
SNP in CNVR	1805	493,241
SNP not in CNVR	10,473	8,114,213

CNVRs were defined as the boundaries identified by GenomeSTRiP plus 5 kb upstream and downstream flanks to maintain consistency with the Tag SNP analysis

556 of the 1805 SNP with significant iHS scores were within 548 genes (+/− 5 kb flanks), including 146 protein coding genes (Additional file 9). The genes were classified by Ensembl Gene Type and the observed numbers of each gene type were compared with expected numbers from Ensembl (Table 4).

**Table 4:** Classification of Genes in CNVR with evidence of selection

Type	Observed Count	Count in Ensembl	Ratio Observed: Expected
pseudogene	259	14,975	1.5
protein_coding	184	21,817	0.7
lincRNA	89	7177	1.1
IG_V_gene	25	138	16.1
IG_V_pseudogene	22	187	10.5
antisense	20	5339	0.3
miRNA	19	3243	0.5
snRNA	11	2001	0.5
processed_transcript	9	799	1.0
IG_C_gene	9	14	57.2
misc_RNA	8	2127	0.3

SNP with evidence of selection were annotated with a gene name if they were within 5 kb of the gene start or end. Counts of gene types were based on Ensembl annotation and the Count in Ensembl was the total number of each type recorded in Ensembl Biomart

Immunoglobulin heavy chain variable and constant region genes were particularly overrepresented with 16 and 57 times as many genes in these classes as would be expected by chance. However, since these genes are found in tight clusters, the counts in CNVRs are not independent and this observation needs interpreting with some caution. Protein coding genes were under-represented with 75% of the expected number.

The mean frequency of CNVs in the CNVRs with SNPs under selection (19%) was twice that of CNVRs without SNPs under selection (8.5%) (χ^2^ = 11,673; *p* < 10^− 10^, Table 5). CNVs may have been driven to higher frequency by selection in these populations.

**Table 5:** Counts of CNV at CNVR with and without SNP under selection

	Deletions	Wild Type	Insertions
CNVR with Selected SNP	2779	39,811	6534
CNVR without Selected SNP	83,003	1,566,194	63,553

There were 2693 CNVRs with SNPs that tag haplotypes in the UNL population and 372 CNVRs with SNPs with evidence of selection. Given that there was a total of 7608 CNVRs, 132 CNVRs would be expected to have both tag SNPs and SNPs with evidence of selection. However, 222 CNVRs were observed with both tag SNPs and SNPs with evidence of selection, more than 50% as many as expected (*p* = 2.8^− 15^) (Additional file 9 and Additional file 10). There was also a 32% excess of individual SNPs that both tagged CNVRs and had evidence of selection (16 expected; 22 observed) but this was not significant (*p* = 0.09).

### Population structure and differentiation

**Fig. 6:**
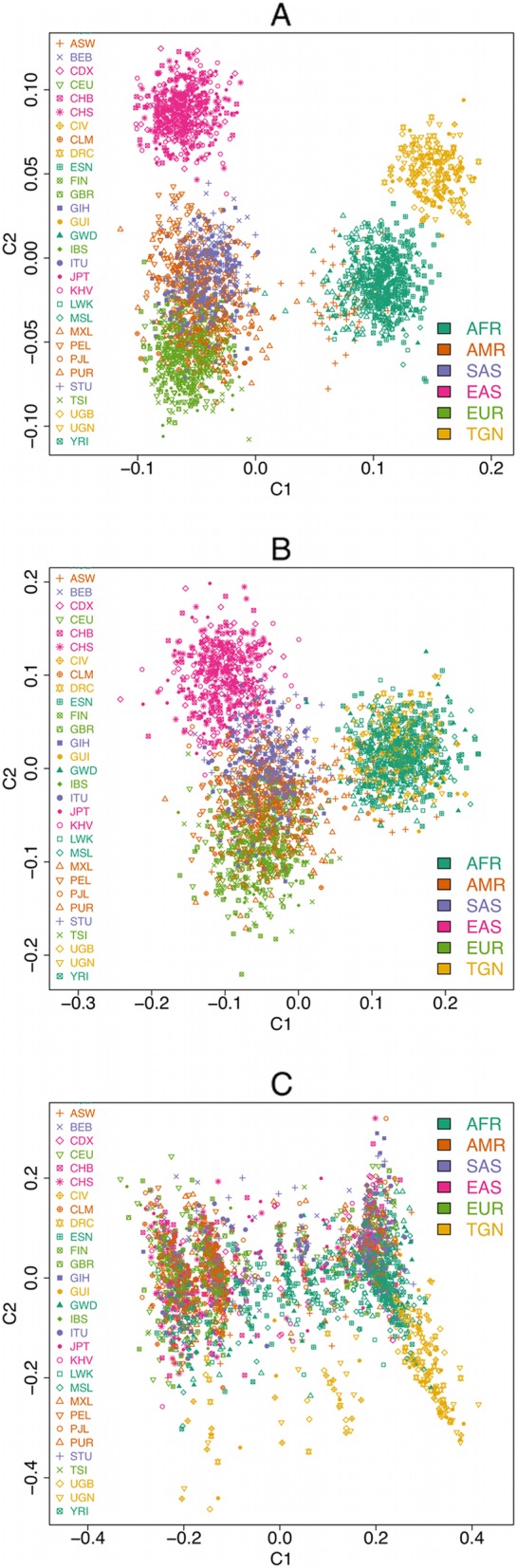
PCA plot showing CNV population structure in our data compared to 1000 Genomes. The PCA distinguishes major continental populations from each other, but is not able to resolve specific populations within the continental populations. Africans in the 1000 Genomes (AFR) are closer to our data (TGN). Conventions for major continental populations are described by the 1000 genomes project [8, 23]. **b** PCA plot showing population structure for bi-allelic deletion CNV. Phase information is non-ambiguous for bi-allelic deletions. The Africans in the 1000 Genomes overlay the TrypanoGEN African samples, indicating similar CNV in the datasets. **c** PCA plot showing population structure due to bi-allelic insertion CNV. There was no specific pattern observed as fewer bi-allelic insertions were available in the data

Principal Component Analysis (PCA) of combined 1000 Genomes and TrypanoGEN populations showed population structure at the continental level (East Asians, South Asians, Caucasians, Americans, Africans) Fig. 6a. However, there was no evidence of structure within most continental populations including Africans (Fig. 6a, b, c). Considering bi-allelic deletions only, the populations in our study here coincided with the 1000 Genomes African populations (Fig. 6b), but bi-allelic duplications revealed no population structure within Africa.

F_ST_ analyses of CNVs showed little difference (F_ST_ < 0.05) between populations (Table 6). The Nilo-Saharan Lugbara from Uganda (UNL) were the most distinctive, F_ST_ between UNL and Niger-Congo populations were approximately double those amongst Niger-Congo populations.

**Table 6:** F_ST_ for CNVs computed from numbers of deletions per locus

	UNL	DRC	GAS	UBB	CIV
**UNL**	0				
**DRC**	0.004	0			
**GAS**	0.008	0.004	0		
**UBB**	0.004	0.003	0.004	0	
**CIV**	0.008	0.004	0.001	0.004	0

F_ST_ were calculated in PLINK using only bi-allelic deletions since phase of these is known

Although the mean F_ST_ across all CNVRs could not distinguish between populations 486 CNVRs show high F_ST_ (> 3 standard deviations from the mean F_ST_) between populations. High F_ST_ loci (> 3sd) intersected selected loci (iHS > 3) within our data. CNVR regions with the highest F_ST_ difference between populations are annotated in Additional file 11. They overlap genes which have been associated with such disease; such as *UGT2B17* (UDP Glucuronosyltransferase Family 2 Member B17) associated with the bone mineral density quantitative trait locus and *IRGM* (Immunity-related GTPase family M protein) associated with inflammatory bowel disease 19.

## Discussion

### CNVR description and novel CNVRs

We identified 7608 consensus CNVs, using GenomeSTRiP and cnMOPS in five African populations. We only retained CNVRs that were called in more than one sample and were identified both by cn.MOPS and GenomeSTRiP. The cn.MOPS CNVRs were much larger, with a mean of 4.5 GenomeSTRiP CNVRs overlapping each cn.MOPS CNVR (Table 2). Given the better match of GenomeSTRiP CNVR size to the DGV CNVR size we interpreted this as evidence that cn.MOPS did not correctly identify CNVR breakpoints and had merged multiple independent CNVRs. cn.MOPS only uses read depth while GenomeSTRiP combines read pairs, split reads, and read depth to generate CNV calls [14]. It is known that the identification of breakpoints is more difficult with read depth dependent methods [24], but the large size difference suggests that cn.MOPS may have been missing breakpoints altogether and concatenating adjacent CNVRs. The differences in CNVs detected by GenomeSTRiP and cn.MOPS are consistent with reports observing that different algorithms for detecting CNVs from whole genome sequencing data show major differences in the CNVs detected [25]. Therefore, to minimise the risk of identifying CNVs at CNVRs that were an artefact of a particular algorithm we used the conservative approach of only identifying CNVs at CNVRs detected by both algorithms.

224 of the 7608 CNVR were defined as novel since they have not been previously submitted to the DGV. All the novel loci had low frequencies of < 10% (Fig. 5a). The locations of CNVR breakpoints are rarely identified very precisely making it difficult to distinguish homologous CNVR from merely overlapping ones [24]. We have taken a conservative approach to defining novel CNVRs by including only those that do not overlap known ones. Given that DGV CNVRs span 75% of the genome and that the remainder includes centromeres and telomeres and repeat regions the low proportion of CNVs in novel regions is not surprising. None of the novel CNVs in our data were common and less than 2% were shared between populations.

### Genomic distribution of CNVR

There was a threefold variation in CNV and CNVR frequency per Mb between chromosomes in our dataset and a nearly twofold variation in the 1000 Genomes data, even after correction for chromosome length (Fig. 3). The density of CNVRs per Mb for each chromosome was correlated in the 1000 Genomes and our datasets (*r*^*2*^ = 0.71), suggesting that CNVR density may be an intrinsic property of chromosomes. The shorter chromosomes tended to have the higher densities of CNVRs in both our data and the 1000 Genomes data and a probe based study of CNVR distribution also found relatively high CNVR density on shorter chromosomes (15,16 and 22) [26]. Although the different studies found different chromosomes with maximum CNVR density, in all cases the highest densities were on the shorter chromosomes, and it is possible that these are more sensitive to structural variation or that shorter chromosomes have higher variance on these parameters. A cross species comparison would be required to test this hypothesis.

### Tagging haplotypes to CNV

CNVs may be the functional variant underlying some QTLs discovered by genome wide association studies using SNP chips. In order to identify SNPs that could predict the presence of CNVs at a locus, we discovered haplotypes with alleles that were associated with CNVs at a locus. The haplotypes we predicted are not only associated with the presence or absence of the CNV locus but also the likely copy number. Previous studies have shown correlations between SNPs or haplotypes and CNVRs. High copy numbers of CNVs at the *HPR* (Haptoglobin related protein) locus have been tagged by haplotypes [27]. There is also strong correlation between alleles of a SNP and CNVs in the *CCL4* (Cysteine-Cysteine Ligand 4) chemokine gene [28].

In the current study, SNP haplotypes tagged 41% of CNVRs. The mean frequency of CNVs at tagged CNVRs (12%) was nearly twice that of untagged loci (7%). This may reflect a lower power to detect associations with rarer CNVs. Longer CNVRs tended to contain more haplotypes and a higher proportion of these were associated with copy number. The relationship between SNP haplotypes and CNVs could be confounded by the same CNV recurring on different haplotypes or by clustering overlapping non-homologous CNV into a single CNVR. The weak association between CNV genotype and population structure in the PCA analysis was consistent with both these hypotheses. Therefore, the number of CNVRs associated with SNP haplotypes may be an indicator of the proportion of stable, non-recurrent homologous, high frequency CNVRs.

### CNVs may be driving selection at some loci

The excess of CNVRs with both tagged haplotypes and SNPs with signatures of selection (−log_10_ p iHS > 3) suggests that the CNV may be the genomic feature that is under selection at these loci. CNVs have been found to be the structure under selection by other methods [29] but this is the first time that we are aware that the combination of SNP signatures of selection combined with SNP haplotype tags have been used. This strategy makes it possible to use the extended haplotype homozygosity test to identify CNVRs under selection, which is more powerful than the previous methods based on F_ST_. However, it should be noted that although there was a highly significant excess of CNVRs with SNPs that tagged CNVs and SNPs that had evidence of selection, the 32% excess of SNPs that were both Tag SNP and had evidence of selection was not significant. Therefore, it is possible that the tag SNP and the SNP with evidence of selection may both be correlated with some third factor other than CNV.

Among the loci that had CNVR overlapping selected loci were Rhesus D (RhD), *C1orf63* (Chromosome 1 Open Reading Frame 63), Human Leukocyte Antigen (*HLA*), Killer-cell Immunoglobulin-like Receptor (*KIR*). The complete deletion of the RhD gene is the commonest cause of Rhesus negative status. Given the severe consequences of the interaction between Rhesus negative (Rh^-ve^) mothers with Rhesus positive (Rh^+ve^) foetus it has been assumed that the null allele might be maintained by some yet unknown selective advantage. Genetic studies have found evidence for heterozygous advantage at the RhD locus in an ecological regression study [30] and an analysis of Rh blood group genes shows that they have experienced positive selection [31]. However, an evolutionary genetics study of the RhD genetics found no evidence for positive natural selection affecting the frequency of the RhD selection [32]. These studies have mostly been conducted in European populations, our study has found evidence of both deletions and insertions at this locus so it is not clear which allele might be under selection.

Variants in the Human Leucocyte Antigen, class II, DQ beta 1 (*HLADQB1*) has been associated with preeclampsia in Iranian women [33]. Interestingly, the *HLA* locus interacts with the locus for *KIR* which has been associated with preeclampsia in Ugandan Bantu women [34]. *KIR3DL1* is also associated with risk of HIV [35]. Given the association of *HLADQB1* and *KIR* in preeclampsia and infectious disease which may impact infant birth and survival, they may be the actual targets of positive selection, resulting in the signatures of selection which have been seen in these loci. These observations generate useful hypothesis for testing. If QTL are discovered in these regions, then CNV should be high priority candidates for the functional variant.

### Population structure

#### WGS CNVs resolve continental populations but not intra-continental populations

We found that CNV distinguish major continental populations, when we included Asians, South Asians, Americans, Europeans and Africans from the 1000 Genomes in the same PCA plot. Similarly, the 1000 Genomes project found CNV data resolve population clustering at continental scales [2]. Consequently, it appears that African populations may not be resolved using CNVR data, although the current study was limited to individuals of Nilo-Saharan, Niger-Congo A and Niger-Congo B origins and did not have access to the Afro-Asiatic or Khoisan populations. To confirm that the inability to resolve populations within Africa was not an artefact of the dataset, we combined our data with 1000 Genomes Project data and found that samples clustered by continent of origin but not at any finer scale. In contrast to these observations Nilo-Saharans, Niger Congo A and Niger Congo B have been shown to cluster separately in SNP based PCA [21]. CNV data therefore have low resolution in distinguishing intra-continental populations despite genomic CNV accounting for at least seven times more genomic base variation than SNP [36]. Several factors may account for the poor resolution of population structure analyses using CNV data including: 1) overlapping but distinct CNVs being coded as from a single CNVRs; 2) samples with different phase being coded the same, e.g. samples with 3:1 chromosomal copies being coded as 2:2; 3) recurrent CNVR at the same locus that do not correlate with population history. The potential importance of phase was illustrated by the better resolution obtained in both the PCA and the F_ST_ analysis using only CNVRs with complete deletions, which means that phase is known. Recurrent CNV at specific CNVR have been identified in different colonies of the same mouse strain and have been associated with disease in humans such as bronchopulmonary dysplasia (BPD) among premature infants [37, 38]. In a study of parent child trios up to 7% of variant loci in the child could not be associated with variants in the parents, which is indicative of novel or recurrent variants or alternatively, problems in variant genotyping [39]. This rate of recurrent mutation could quickly disrupt associations between variant genotypes and populations.

#### Bias of selection in CNVR despite low F_ST_

Consistent with the PCA analysis, global F_ST_ showed that African CNV frequencies were similar across populations. However, there were 3–5-fold more CNVRs than expected by chance in regions where previous studies have found evidence for selection (*p* < 0.00001). The higher than expected number of CNVRs in regions under selection suggests either the CNVRs are under selection or that selection signatures are more likely to arise in CNVRs regions. Consistent with the hypothesis that selection drives the enrichment of CNVRs in particular populations, the alpha thalassaemia deletion reveals signatures of selection [40] and is selected to high frequencies in malaria endemic areas because it confers protection against infection by the malaria-causing parasite *Plasmodium* [41] and in our data the *KIR* locus had both SNP signatures of selection and SNP haplotypes tagging CNV. This suggests that CNVs are the polymorphism under selection in at-least some of these regions.

## Conclusion

We have presented a CNVR landscape of populations representing the Niger-Congo A, Niger-Congo B and Nilo-Saharan African ethnic groups. These include known CNVRs that have been described in the DGV, and novel ones (3%), that are not reported in the DGV, reflecting the diverse nature of these African populations. Some of the CNVRs described may have medical significance as they occur in Mendelian disease-causing genes and overlap SNPs significantly associated with various traits in the GWAS catalogue. We have used haplotypes to tag CNVRs. Haplotypes tagging CNVRs are useful in imputing CNVs from SNP genotyping data in future studies, especially in African populations known to have low linkage disequilibrium. We found overrepresentation of CNVRs in regions showing signatures of selection in SNP based studies, and an excess of CNVRs with both haplotypes tagging CNVs and SNP haplotypes with signatures of selection, suggesting a possible role of CNVR in selection and adaptation. Finally, we show that CNV distinguish between continental populations but do not stratify within the continent, such as the Africans in the current study.

## Materials and methods

### Population description

The study was conducted in the context of the TrypanoGEN project [42], which aims to determine host genetic susceptibility to Human African Trypanosomiasis. Samples were selected from the TrypanoGEN bio-bank [42]. The populations included were from East, Central and West Africa. East African populations were the Ugandan Nilo-Saharan language speakers (Lugbara) (*n* = 50) from Northern Uganda and Ugandan Niger-Congo B speakers (Basoga) (*n* = 33) whereas Central African populations were Niger-Congo B speakers (n = 50) from the Democratic Republic of the Congo. West African populations were Niger-Congo A speakers (*n* = 49) from Guinea and Cote D’Ivoire (n = 50). The samples in the current study are a subset of those described in the TrypanoGEN bio-bank [42]. Sample collection, ethical considerations and approvals have been previously described [42]*.* The summary of the population, linguistic group and sampling foci are in Table 1.

### Sequencing and SNP calling

We used the Illumina Truseq polymerase chain reaction (PCR) free kit to prepare WGS libraries. Sequencing was done at the Centre for Genomic Research at the University of Liverpool using the Illumina Hiseq2500 system at 10X coverage. We used Burrow Wheeler Alignment (BWA) to map sequenced reads onto the 1000 Genomes project human_g1k_v37_decoy reference genome. The Genome analysis tool kit (GATK v3.4) was used for SNP calling following GATK best practice guidelines. Quality control measures of SNPs included filtering by a) removing loci with > 10% missing SNP, b) removing individuals with > 10% missing SNP loci and c) removing loci with Hardy Weinberg *P*-value < 0.01.

### CNV description and functional analysis

We use CNVR to refer to a locus at which one or more samples may have a CNV; the overlapping CNVs at a CNVR may each have different boundaries. To select methods to identify CNVR we reviewed the literature and found 4 major approaches to CNV discovery: 1) Paired end methods (PE) estimate insert size between the paired ends but is limited by the size of the fragment sequenced; 2) Split Read methods (SR) are focused on identifying exact break points in reads that do not map but have a mate pair that maps perfectly, it works well with deletions but can only identify small (< 50 bp) insertions; 3) Read Depth (RD) methods count the number of reads at each genomic location and 4) De Novo assembly methods create new genome assemblies and compare them with the reference and are best suited to high coverage with long read data [14]. Read Depth methods are most widely used for CNV discovery as they can estimate numbers of copies whereas PE and SR methods detect the presence of a variant but cannot quantify it. RD methods can be further subdivided into those that use a single sample, paired samples (e.g. parent child) or population samples. Of the six methods benchmarked recently by Trost and colleagues [43], only cn.MOPS and GenomeSTRiP use population scale data. Ideally, the performance of these algorithms should be evaluated against ‘known’ CNVR. This reference of ‘known’ CNVR is made when several algorithms are used to come up with consensus CNVR. To evaluate performance of algorithms, the results of each algorithm are compared to the consensus ‘known’ CNVR by calculating sensitivity (proportion of CNV in benchmark which an algorithm identifies) and false discovery rate (proportion of CNVs discovered by the algorithm that are not in the benchmark). Due to limited African CNV datasets, we referenced an evaluation of CNVR detection algorithms for sensitivity and false discovery rate against CNVR in the HuRef CNV Benchmark [43]. From these evaluations, two algorithms (GenomeSTRiP and cn.MOPS) integrated data from multiple samples concurrently to discover CNVR and showed reasonable sensitivity and false discovery rates. GenomeSTRiP had sensitivity of 0.68; and a false discovery rate of 0.49, whereas cn.MOPS had sensitivity of 0.38; and a false discovery rate of 0.33. We therefore used GenomeSTRiP [27] and cn.MOPS [44] to detect CNVs in binary alignment map (BAM) files of our data. GenomeSTRiP has previously been used to detect CNVs in the 1000 Genomes project of human populations [27]. To validate detected CNVs we tested for overlap with published CNVs in the public Database of Genomic Variants (DGV; release date 2016-05-15) using BEDTools [18]. For GenomeSTRiP we used parameters recommended for 10X sequence data, whereas for cn.MOPS, we tested various parameters (Additional file 12). We annotated CNV overlaps with gene names from the UCSC genome browser [45, 46] and Ensembl Biomart [47] for Genome version hg19/GRCh37 using BEDTools [18].

### Population clustering (PCA)

We sought evidence for population structure due to CNVs using PCA in PLINK [48]. CNV data were first converted into multi-allelic genotype format and represented as 1 1 (0 copies), 1 2 (1 copy), 2 2 (normal copy number 2), 2 3 (3 copies), 3 3 (4 copies) up to a maximum of 4 4 for six copies of more. Since phase was not known we assumed that alleles were as equally distributed between chromosomes as possible. To merge with 1000 genomes data, common loci in both datasets were used. PLINK was used for population clustering as described in the documentation. We used the PLINK cluster command, which relies on identity by state values and Hamming distance to perform complete linkage clustering. R was used to visualise the resulting principal components.

### Population differentiation: F_ST_ analysis

We investigated population differentiation by comparing F_ST_ between CNVs in the different populations. We used multi-allelic data format as described above for population differentiation (F_ST_) analysis. F_ST_ were calculated using PLINK v 1.9 [48] using only bi-allelic deletions since phase of these is known.

### Tag haplotypes for CNV

We used the method described by Handsaker et al. [13], implemented with a custom Java programme [49]. This method assumes that if a haplotype is associated with a CNV then the number of alleles (0,1,2) of that haplotype will be correlated with the observed number of copies reported for each sample. Therefore, copy number is plotted against haplotype count and the value of *r*^*2*^ is calculated for the regression line and the *p* value for the null hypothesis that the slope of the regression line is zero. We used a custom Java program; TagCNV available from Github [49] to find SNP haplotypes and test their association with CNVs. To build haplotypes VCFtools was used to obtain the correlation (r^2^) between alleles in 50 kb windows. Sets of alleles within 5 kb of CNV boundaries and with r^2^ > 0.8 with at least one other SNP in the region were assembled into haplotypes. The counts of each allele were plotted against the fractional copy number for the CNV for each sample and the correlation (*r*^*2*^) between haplotype count and copy number was obtained using R and the association was considered significant if *p* < 0.05 for the null hypothesis that the slope of the regression line is zero. A Bonferroni correction was applied for the number of haplotypes at the CNVR that were tested for association. Haplotypes which were only present in samples with identical copy numbers were considered uninformative and excluded from all calculations.

## Supplementary information

**Additional file 1: Table S1.** List of samples in the study.

**Additional file 2.** Correlation of GenomeSTRiP and cn.MOPS and supplementary figures.

**Additional file 3: Table S2.** GenomeSTRiP CNVR that intersect cn.MOPS CNVR after QC.

**Additional file 4: Table S3.** Counts of CNV types detected by both algorithms.

**Additional file 5: Table S4.** Genes intersected by novel CNV.

**Additional file 6: Table S5.** Novel CNVR intersecting the GWAS catalogue SNPs.

**Additional file 7: Table S6A.** All CNVR intersecting OMIM genes B. Novel CNVR intersecting OMIM genes.

**Additional file 8: Table S7.** Haplotypes that tag CNVR in each of the populations.

**Additional file 9: Table S8.** Counts of CNVR and SNP with tagged SNP and SNP with Signatures of Selection (iHS > 3).

**Additional file 10: Table S9.** CNVR tagged by SNP haplotypes and also containing SNP signatures of selection.

**Additional file 11: Table S10.** CNVR with high F_ST_ between populations.

**Additional file 12: Table S11.** Effect of Prior Impact and Minimum width on concordance with DGV in cn.MOPS.

## Data Availability

The sequences dataset used in this study is available from the European Genome-phenome Archive (EGA): EGAS00001002602). The program for tagging haplotypes to CNVR, TagHap.jar is available from Github [49]. GenomeSTRiP was downloaded from the GenomeSTRiP website [50]. cn.MOPS is also available from bio-conductor [51].

## Abbreviations

BAM: Binary Alignment Map
BCI: Baoule ethnologue code
BWA: Burrow Wheeler Alignment
*C1orf63*: Chromosome 1 Open Reading Frame 63
*CCL4*: Cysteine-Cysteine Ligand 4 chemokine gene
CIV: Côte d’Ivoire Niger Congo A speakers
CM: cn.MOPS
cn.MOPS: Copy number mixture of Poissons
CNV(s): Copy number variant(s)
CNVR(s): Copy number variant region(s)
DGV: Database of genomic variants
DRC: Democratic Republic of Congo
F_ST_: F statistics
GAS: Guinea Niger Congo A speakers
GATK: Genome Analysis Tool Kit
GenomeSTRiP: Genome Structure in Populations
GS: GenomeSTRiP
GO: Gene ontology
GOA: Gouro ethnologue code
GWAS: Genome wide Association study
HIV: Human Immunodeficiency Virus
H3Africa: Human Heredity and Health in Africa
*HLADQB1*: Human Leucocyte Antigen, class II, DQ beta 1
*HPR*: Haptoglobin related protein
IGG: Lugbara ethnologue code
iHS: Integrated haplotype score
*IRGM*: Immunity-related GTPase family M protein
KEMRI: Kenya Medical Research Institute
KGA: Koyaka ethnologue code
*KIR*: Killer-cell Immunoglobulin like receptor
*KIR3DL1*: Killer cell immunoglobulin-like receptor 3DL1
LOI: Malinke ethnologue code
MDP: Kingongo ethnologue code
MDS: Multidimensional scaling
MOA: More ethnologue code
NCA: Niger Congo A
NCB: Niger Congo B
NOQ: Kimbala ethnologue code
NS: Nilo Saharans
PCR: Polymerase chain reaction
PE: Paired end CNV detection methods
QC: Quality control
QTL: Quantitative Trait Loci
RD: Read depth
RhD: Rhesus D
SEF: Senoufo ethnologue code
SNP(s): Single Nucleotide Polymorphism(s)
SR: Split read CNV detection methods
SUS: Soussou ethnologue code
UBB: Uganda Bantu Basoga
UCSC: University of California Santa Cruz
*UGT2B17*: UDP Glucuronosyltransferase Family 2 Member B17
UNL: Uganda Nilotic Lugbara
WGS: whole genome sequence
XOG: Basoga ethnologue code

## References

[1] Redon R, Ishikawa S, Fitch KR, Feuk L, Perry GH, Andrews TD (2006). Global variation in copy number in the human genome. Nature..

[2] Sudmant PH, Mallick S, Nelson BJ, Hormozdiari F, Krumm N, Huddleston J (2015). Global diversity, population stratification, and selection of human copy-number variation. Science.

[3] Sudmant PH, Rausch T, Gardner EJ, Handsaker RE, Abyzov A, Huddleston J (2015). An integrated map of structural variation in 2,504 human genomes. Nature..

[4] Gamazon ER, Stranger BE (2015). The impact of human copy number variation on gene expression. Brief Funct Genomics.

[5] Perry GH, Dominy NJ, Claw KG, Lee AS, Fiegler H, Redon R (2007). Diet and the evolution of human amylase gene copy number variation. Nat Genet.

[6] Hollox EJ, Hoh B-P (2014). Human gene copy number variation and infectious disease. Hum Genet.

[7] Lee C, Scherer SW (2010). The clinical context of copy number variation in the human genome. Expert Rev Mol Med.

[8] Auton A, Brooks LD, Durbin RM, Garrison EP, Kang HM, 1000 Genomes Project Consortium (2015). A global reference for human genetic variation. Nature.

[9] Tishkoff SA, Reed FA, Friedlaender FR, Ehret C, Ranciaro A, Froment A (2009). The genetic structure and history of Africans and African Americans. Science..

[10] Gurdasani D, Carstensen T, Tekola-Ayele F, Pagani L, Tachmazidou I, Hatzikotoulas K (2014). The African genome variation project shapes medical genetics in Africa. Nature..

[11] Inchley CE, Larbey CDA, Shwan NAA, Pagani L, Saag L, Antão T (2016). Selective sweep on human amylase genes postdates the split with Neanderthals. Sci Rep.

[12] Matovu E, Bucheton B, Chisi J, Enyaru J, Hertz-Fowler C, The H3Africa Consortium (2014). Enabling the genomic revolution in Africa. Science.

[13] Eberhard DM, Gary FS, Charles DF, (eds). Ethnologue: Languages of the World. Twentythird edition. 2020. https://www.ethnologue.com/. Accessed 20 Mar 2020.

[14] Zhao M, Wang Q, Wang Q, Jia P, Zhao Z (2013). Computational tools for copy number variation (CNV) detection using next-generation sequencing data: features and perspectives. BMC Bioinformatics.

[15] Wright S. Coefficients of inbreeding and relationship. Am Nat. 1922;56:330–8.

[16] MacDonald JR, Ziman R, Yuen RKC, Feuk L, Scherer SW (2014). The database of genomic variants: a curated collection of structural variation in the human genome. Nucleic Acids Res.

[17] DGV. Database of Genomic Variants. 2017. http://dgv.tcag.ca/dgv/docs/Inclusive.Gain+Loss.hg19.2015-02-03.txt. Accessed 5 Jul 2017.

[18] Quinlan AR, Hall IM (2010). BEDTools: a flexible suite of utilities for comparing genomic features. Bioinforma Oxf Engl.

[19] PANTHER - Gene List Analysis. http://www.pantherdb.org/. Accessed 5 Jul 2019.

[20] Shifman S, Kuypers J, Kokoris M, Yakir B, Darvasi A (2003). Linkage disequilibrium patterns of the human genome across populations. Hum Mol Genet.

[21] Mulindwa J, Noyes HA, Ilboudo H, Nyangiri O, Koffi M, Mumba D, et al. Evidence of population specific selection inferred from 289 genome sequences of Nilo-Saharan and Niger-Congo linguistic groups in Africa. bioRxiv. 2017. 10.1101/186700.

[22] Nguyen D-Q, Webber C, Ponting CP (2006). Bias of selection on human copy-number variants. PLoS Genet.

[23] Population | 1000 Genomes. https://www.internationalgenome.org/category/population/. Accessed 27 Feb 2020.

[24] Abyzov A, Urban AE, Snyder M, Gerstein M (2011). CNVnator: an approach to discover, genotype, and characterize typical and atypical CNVs from family and population genome sequencing. Genome Res.

[25] Pabinger S, Dander A, Fischer M, Snajder R, Sperk M, Efremova M (2014). A survey of tools for variant analysis of next-generation genome sequencing data. Brief Bioinform.

[26] Kato M, Kawaguchi T, Ishikawa S, Umeda T, Nakamichi R, Shapero MH (2010). Population-genetic nature of copy number variations in the human genome. Hum Mol Genet.

[27] Handsaker RE, Van Doren V, Berman JR, Genovese G, Kashin S, Boettger LM (2015). Large multiallelic copy number variations in humans. Nat Genet.

[28] Colobran R, Comas D, Faner R, Pedrosa E, Anglada R, Pujol-Borrell R (2008). Population structure in copy number variation and SNPs in the CCL4L chemokine gene. Genes Immun.

[29] Iskow RC, Gokcumen O, Lee C (2012). Exploring the role of copy number variants in human adaptation. Trends Genet TIG.

[30] Flegr J (2016). Heterozygote advantage probably maintains rhesus factor blood group polymorphism: ecological regression study. PLoS One.

[31] Kitano T, Saitou N (1999). Evolution of Rh blood group genes have experienced gene conversions and positive selection. J Mol Evol.

[32] Perry GH, Xue Y, Smith RS, Meyer WK, Calışkan M, Yanez-Cuna O (2012). Evolutionary genetics of the human Rh blood group system. Hum Genet.

[33] Mohammadi M, Farazmandfar T, Shahbazi M (2017). Relationship between human leukocyte antigen (HLA)-DQA1*0102/HLA-DQB1*0602 polymorphism and preeclampsia. Int J Reprod Biomed Yazd Iran.

[34] Nakimuli A, Chazara O, Hiby SE, Farrell L, Tukwasibwe S, Jayaraman J (2015). A KIR B centromeric region present in Africans but not Europeans protects pregnant women from pre-eclampsia. Proc Natl Acad Sci U S A.

[35] Pelak K, Need AC, Fellay J, Shianna KV, Feng S, Urban TJ (2011). Copy number variation of KIR genes influences HIV-1 control. PLoS Biol.

[36] Conrad DF, Pinto D, Redon R, Feuk L, Gokcumen O, Zhang Y (2010). Origins and functional impact of copy number variation in the human genome. Nature..

[37] Egan CM, Sridhar S, Wigler M, Hall IM (2007). Recurrent DNA copy number variation in the laboratory mouse. Nat Genet.

[38] Ahmad A, Bhattacharya S, Sridhar A, Iqbal AM, Mariani TJ (2016). Recurrent copy number variants associated with bronchopulmonary dysplasia. Pediatr Res.

[39] Chaisson MJP, Sanders AD, Zhao X, Malhotra A, Porubsky D, Rausch T (2019). Multi-platform discovery of haplotype-resolved structural variation in human genomes. Nat Commun.

[40] Qiu Q-W, Wu D-D, Yu L-H, Yan T-Z, Zhang W, Li Z-T (2013). Evidence of recent natural selection on the southeast Asian deletion (−-(SEA)) causing α-thalassemia in South China. BMC Evol Biol.

[41] Flint et al. High frequencies of alpha-thalassaemia are the result of natural selection by malaria. - PubMed - NCBI. https://www.ncbi.nlm.nih.gov/pubmed/3713863. Accessed 27 Mar 2019. 10.1038/321744a0 3713863

[42] Ilboudo H, Noyes H, Mulindwa J, Kimuda MP, Koffi M, Kaboré JW (2017). Introducing the TrypanoGEN biobank: a valuable resource for the elimination of human African trypanosomiasis. PLoS Negl Trop Dis.

[43] Trost B, Walker S, Wang Z, Thiruvahindrapuram B, MacDonald JR, Sung WWL (2018). A comprehensive workflow for read depth-based identification of copy-number variation from whole-genome sequence data. Am J Hum Genet.

[44] Klambauer G, Schwarzbauer K, Mayr A, Clevert D-A, Mitterecker A, Bodenhofer U (2012). cn.MOPS: mixture of Poissons for discovering copy number variations in next-generation sequencing data with a low false discovery rate. Nucleic Acids Res.

[45] Kent WJ, Sugnet CW, Furey TS, Roskin KM, Pringle TH, Zahler AM (2002). The human genome browser at UCSC. Genome Res.

[46] Tyner C, Barber GP, Casper J, Clawson H, Diekhans M, Eisenhart C (2017). The UCSC genome browser database: 2017 update. Nucleic Acids Res.

[47] Cunningham F, Achuthan P, Akanni W, Allen J, Amode MR, Armean IM (2019). Ensembl 2019. Nucleic Acids Res.

[48] Purcell S, Neale B, Todd-Brown K, Thomas L, Ferreira MAR, Bender D (2007). PLINK: a tool set for whole-genome association and population-based linkage analyses. Am J Hum Genet.

[49] Noyes H. Tag Copy Number Variations (CNV) with SNP haplotypes. 2018. https://github.com/LiverpoolHarry/TagCNV. Accessed 2 May 2018.

[50] Genome STRiP | GenomeSTRiP. http://software.broadinstitute.org/software/genomestrip/. Accessed 5 Jul 2019.

[51] cn.mops. Bioconductor. http://bioconductor.org/packages/cn.mops/. Accessed 5 Jul 2019.

