# Supplementary material for: Copy number variation in human genomes from three major ethno-linguistic groups in Africa
Source: BMC Genomics. 2020 Apr 10;21:289. doi: 10.1186/s12864-020-6669-y (PMC7147055; doi:10.1186/s12864-020-6669-y)
Supplement: Supplementary file 2 — Additional file 2. Correlation of GenomeSTRiP and cn.MOPS and supplementary figures. [file 12864_2020_6669_MOESM2_ESM.doc]

**Additional data File 2**

Contents

[Correlation of cn.MOPS and GenomeSTRiP CNV 1](#__RefHeading___Toc33697668)

[Table 1. Numbers of CNVR where there was a significant (p<0.05) association between samples identified as having CNV by GenomeSTRiP and cn.MOPS. 2](#__RefHeading___Toc33697669)

[Table 2. Number of CNV called by GenomeSTRiP (GS) and cn.MOPS (CM). 3](#__RefHeading___Toc33697670)

[Table 3 Numbers of shared CNV at CNVR where GenomeSTRiP (GS)and cn.MOPS have the same and opposite directions of effect. 3](#__RefHeading___Toc33697671)

[List of supplementary figures 4](#__RefHeading___Toc33697672)

[Fig S1 A Distribution of numbers of GenomeSTRiP CNVR under each cn.MOPS CNVR. 4](#__RefHeading___Toc33697673)

[Fig S1 B Distributions of lengths of cn.MOPS and GenomeSTRiP CNVR. 5](#__RefHeading___Toc33697674)

[Fig S2: Multidimensional scaling plot visualizing the spread of raw CNV data. 6](#__RefHeading___Toc33697675)

[Fig S3: Density of CNVR varies by about two-fold in the five populations. 7](#__RefHeading___Toc33697676)

[Fig S4 A: Annotation of CNVR by Ensembl intersects 8](#__RefHeading___Toc33697677)

[Fig S4 B: Comparison of proportions of CNVR overlaps in novel compared to all CNVR. 9](#__RefHeading___Toc33697678)

[Fig S5: CNVR overlap SNP associated with significant traits in the GWAS catalogue. 10](#__RefHeading___Toc33697679)

[Fig S6: Distribution of Tagged and Untagged CNVR by CNV Count. 11](#__RefHeading___Toc33697680)

# Correlation of cn.MOPS and GenomeSTRiP CNV

The mean length of cn.MOPS CNVR (542kb) was much longer than the GenomeSTRiP CNVR (9kb) (Fig 1B below) making it difficult to compare the properties of the CNV called by the two methods. If the two methods were consistent then they would both call the same CNV genotypes in the same samples. We compared consistency using two separate metrics: 1) consistency in the identity of samples that were identified as having CNV at a CNVR; 2) consistency of copy number called at a CNVR.

**Consistency of samples with CNV calls by each method.**

Each cn.MOPS locus included between 1 and 188 GenomeSTRiP strip CNVR (Fig 1A below), this makes it difficult to measure consistency as the relevant CNVR to compare are not easy to identify. We compared CNV at every CNVR identified by GenomeSTRiP at each cn.MOPS locus. The correlation between the two sets of samples was evaluated using a Fisher Exact test. Of the 829 cn.MOPS CNVR with three or less samples with a CNV call there were only 15 CNVR where concordance with GenomeSTRiP was greater than would be expected by chance. However of the 853 CNVR with four or more samples with a CNV call in the cn.MOPS data 574 CNVR (67%) had greater concordance with GenomeSTRiP calls than would be expected by chance (Table1). Consequently, at most common CNVR, there was significant correlation between the two methods in the samples identified.

|  | <=3 CN samples | >=4 CN samples |
| --- | --- | --- |
| Significantly more samples in common than expected by chance | 15 (2%) | 574 (67%) |
| No more samples in common than expected by chance | 814 (98%) | 279 (33%) |

Table 1 Numbers of CNVR where there was a significant (p<0.05) association between samples identified as having CNV by GenomeSTRiP and cn.MOPS. The CNVR are divided based on the number of samples with CNV in the cn.MOPS data.

At 73% of CNVR where cn.MOPS had >=4 samples with CNV (CNV frequency > 2%), more samples had a CNV by both cn.MOPS and GenomeSTRiP than would be expected by chance (p<0.05). However only 46% of samples that had CNV by GenomeSTRiP also had CNV by cn.MOPS at the same CNVR (Table2). This is not surprising given that GenomeSTRiP CNV were much smaller than cn.MOPS CNVR and a mean of 5.9 GenomeSTRiP CNVR were within each cn.MOPS locus. GenomeSTRiP reported 56% more CNV than cn.MOPS; given that it is impossible for these excess CNV to be correlated with cn.MOPS CNVR the consistency of cn.MOPS CNVR with GenomeSTRiP (67%) is perhaps the best indicator of the overall consistency of the two methods.

| Table 2 | CNV only in GS | CNV in GS and CM | CNV only in CM |
| --- | --- | --- | --- |
| CNVR with >= 4 cn.MOPS samples | 26,631(54%) | 22,348 (46% of GS; 73% of CM) | 8,104(27%) |
| CNVR with <= 3 cn.MOPS samples | 6,664 (86%) | 1,081 (14% of GS; 87% of CM) | 161(13%) |

Table 2 Number of CNV called by GenomeSTRiP (GS) and cn.MOPS (CM).

**Consistency of number of copies at each CNV**

Given the large size difference between CNV called between GenomeSTRiP and cn.MOPS it is difficult to compare the effects called by each method. If the same sample has a CNV called by GenomeSTRiP in several CNVR that are all within a single cn.MOPS CNVR what is the best measure of correlation? 57% of CNV that were common to GenomeSTRiP and cn.MOPS were deletions (copy number loss) and 43% were insertions (copy number gain) (Table 2 main text). We have used the average copy number across all GenomeSTRiP CNVR within each cn.MOPS CNVR as the observed phenotype and compared the direction of effect (copy number loss or gain) obtained for each sample at each CNVR (Table 3). There were significantly more gains in both sets or losses in both sets than would be expected by chance (15,285 out 18,093 CNV (84%)) (χ = 3,703; p = 0)(Table3).

|  | CM < 2 copies | CM > 2 copies |
| --- | --- | --- |
| GS < 2 copies | 14,033 | 2,510 |
| GS > 2 copies | 298 | 1,252 |

Table 3 Numbers of shared CNV at CNVR where GenomeSTRiP (GS)and cn.MOPS have the same and opposite directions of effect.

# Supplementary figures

**Distribution of CNVs**

**Fig S1 A Distribution of numbers of GenomeSTRiP CNVR under each cn.MOPS CNVR***.* Over 1,000 cn.MOPS CNVRs have less than three GenomeSTRiP CNVRs under them but about 200 have over 20 and some have over 100.

## Fig S1 B Distributions of lengths of cn.MOPS and GenomeSTRiP CNVR.

GenomeSTRiP CNVR are skewed to much shorter lengths than cn.MOPS CNVR


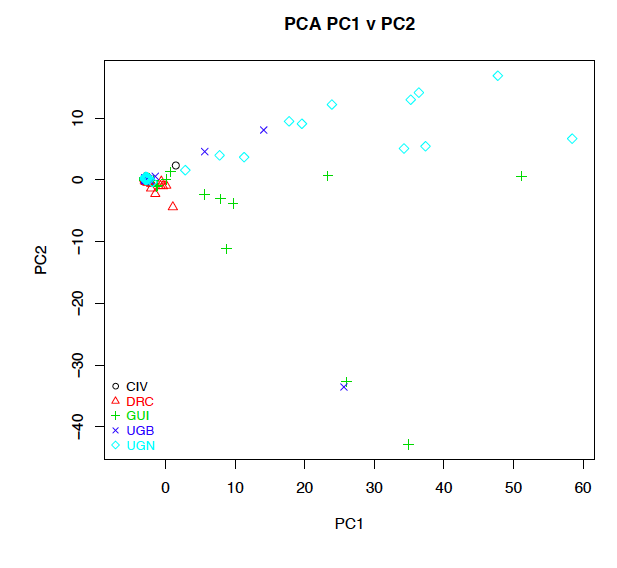


## Fig S2: Multidimensional scaling plot visualizing the spread of raw CNV data.

High scatter samples were identified and excluded from further analysis as they skew results of CNV analysis


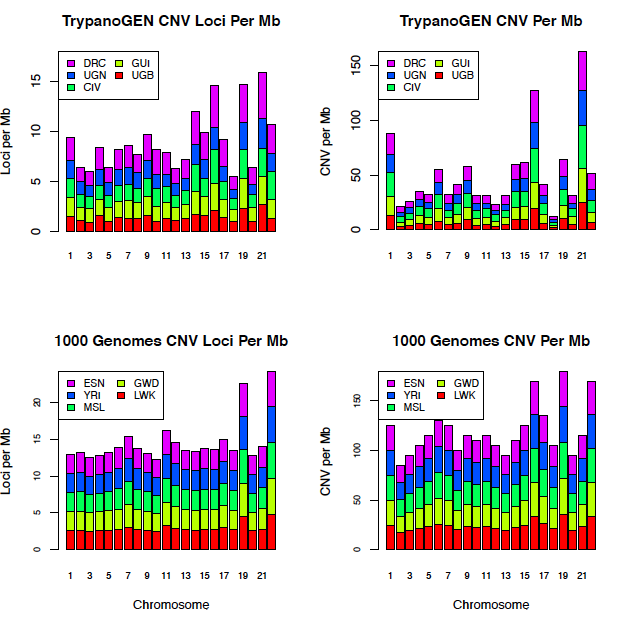


## Fig S3: Density of CNVR varies by about two-fold in the five populations.

There is also CNVR differences by population. The 1000 genomes project samples show the same pattern of variation by population and samples


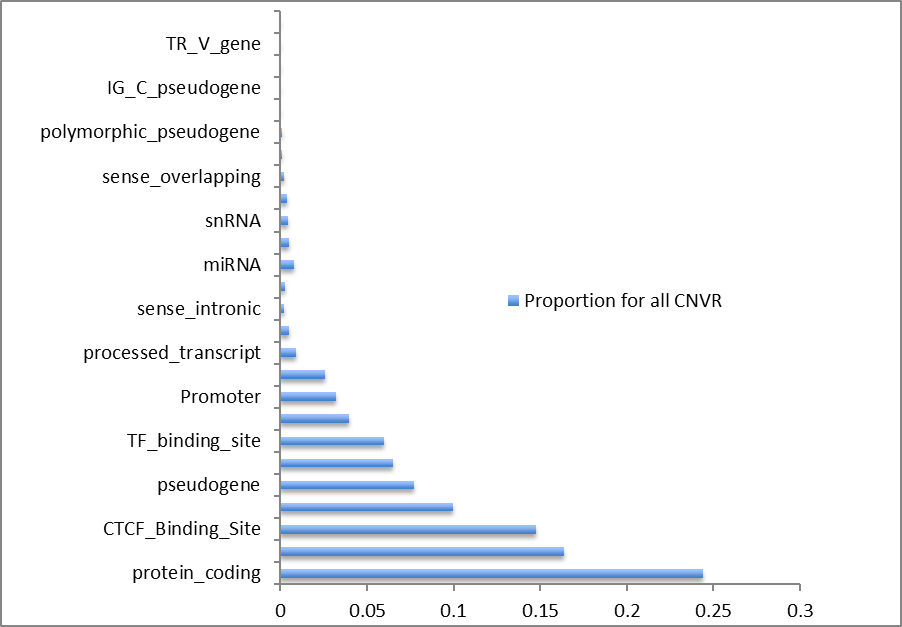


## Fig S4 A: Annotation of CNVR by Ensembl intersects

(A) Annotated CNVR for the whole genome span most of the genomic features, with protein coding, open chromatin and flanking sites having the highest proportions


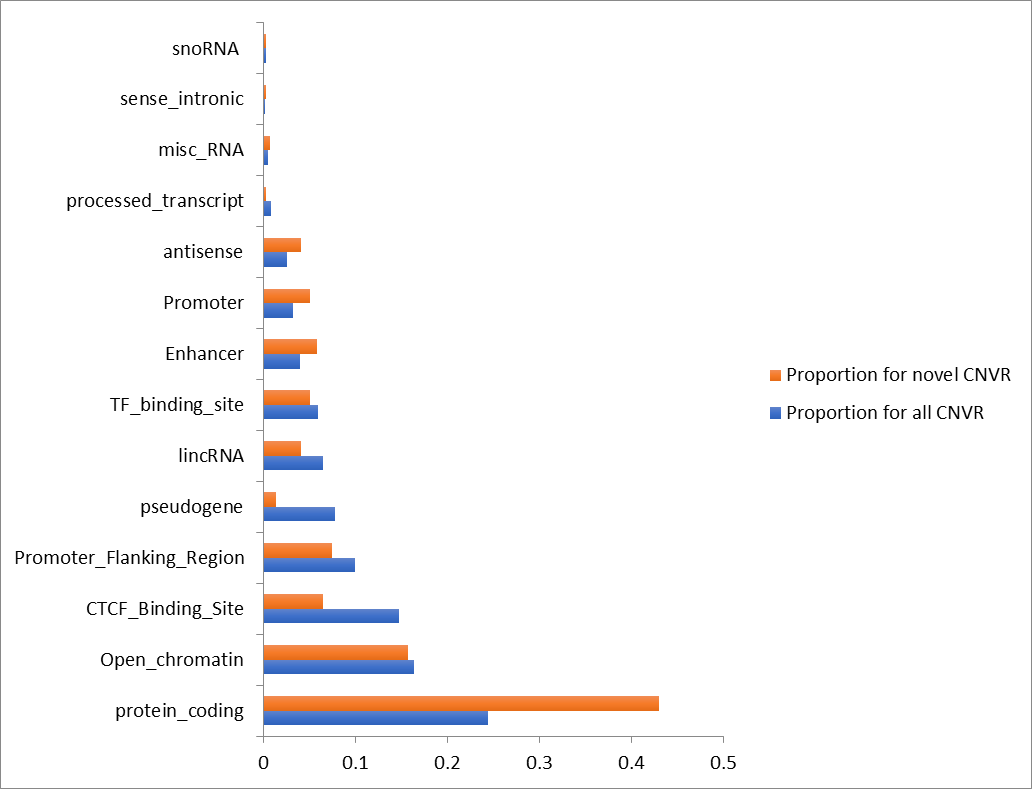


## Fig S4 B: Comparison of proportions of CNVR overlaps in novel compared to all CNVR.

Protein coding regions are more represented in novel CNVR compared to known CNVR, while the proportions of other genomic features are similar. The lowest 12 proportions of the known CNVR (see S4 figure A) had no corresponding novel CNVR discovered and they are therefore not shown here. These are 3prime_overlapping_ncrna, TR_V_gene, IG_D gene, IG_C_pseudogene, IG_C_gene, polymorphic_pseudogene, rRNA, sense_overlapping, IG_V_gene, snRNA, IG_V_pseudogene and miRNA


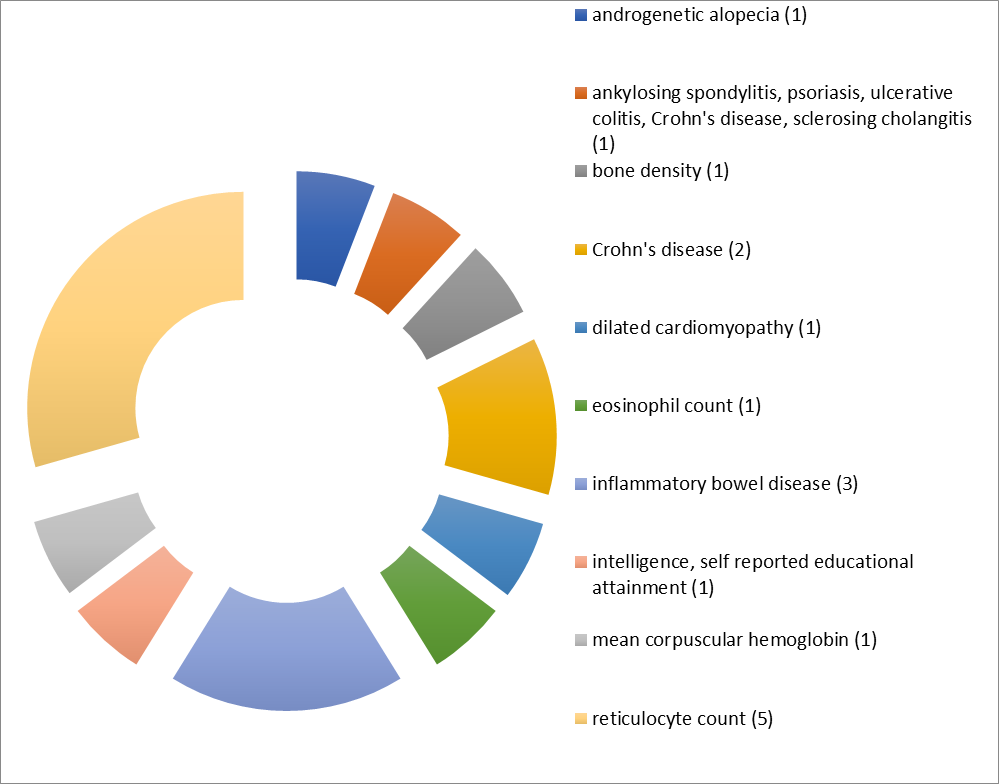


## Fig S5: CNVR overlap SNP associated with significant traits in the GWAS catalogue.

The doughnut segment’s size represents the number of significant SNP in the GWAS catalogue that CNVR overlap. The actual number of SNP is shown in brackets against the trait.


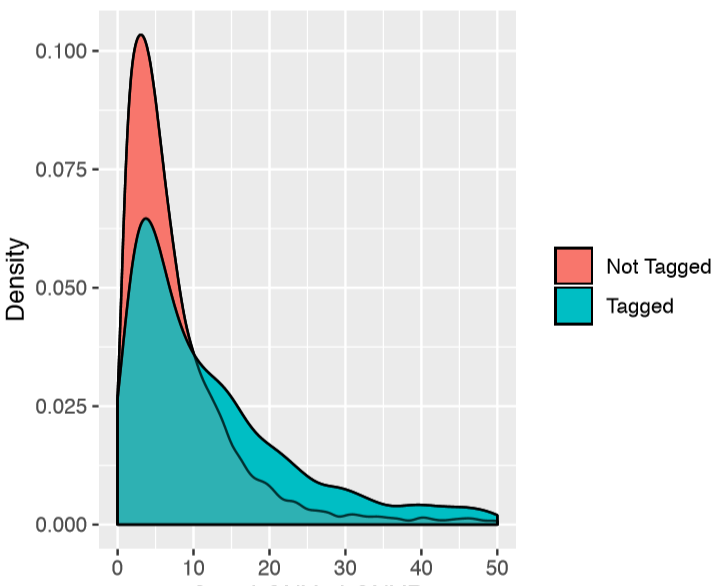


## Fig S6: Distribution of Tagged and Untagged CNVR by CNV Count.

There are more untagged (pink) than tagged (green) CNVR where CNVR have fewer than 12 CNV and more tagged than untagged where CNVR have greater than 12 CNV. The plot is truncated at 50 CNV per CNVR.
